# Hunter Syndrome: The Phenotype of a Rare Storage Disease

**DOI:** 10.7759/cureus.21985

**Published:** 2022-02-07

**Authors:** Rute Sousa Martins, Sara Rocha, Arlindo Guimas, Rosa Ribeiro

**Affiliations:** 1 Internal Medicine, Centro Hospitalar Universitário do Porto, Porto, PRT

**Keywords:** dysostosis multiplex, mucopolysaccharidosis, hunter syndrome, inborn errors of metabolism, rare diseases

## Abstract

Hunter syndrome is a rare lysosomal storage disorder with systemic involvement that occurs over time. Affected patients have coarse facial features, growth retardation with short stature, and skeletal deformities called dysostosis multiplex; joint stiffness, progressive mental retardation, and organomegaly are some of the clinical signs. It ranges from mild to severe manifestations and the distinction between them is related to neurological involvement. Cardiac and respiratory failure is commonly the cause of early death (before adulthood) for severe forms, but those with attenuated forms who have normal cognitive development can survive until late adulthood. Treatment with enzyme replacement therapy is available and can improve the prognosis of this disease. The authors present a case of a 36-year-old male with Hunter syndrome to show not only the clinical features typical of this multisystemic disease that should alert to a prompt investigation but also to remind that treatment must start as early as possible to reach the best outcome. Management of this disease is typically challenging and requires a multidisciplinary approach.

## Introduction

Hunter syndrome or mucopolysaccharidosis (MPS) type II is a rare, X-linked metabolic disease with an estimated prevalence at birth in Europe of 1/166,000 [1]. This disease belongs to the group of lysosomal storage disorders [2-4]. MPS type II occurs due to a deficiency in the activity of iduronate-2-sulfatase, a lysosomal enzyme, which leads to consequent accumulation of mucopolysaccharides, also called glycosaminoglycans (GAGs) [5,6]. As a result, multiple organs are gradually affected as GAGs accumulate over time.

Patients with MPS type II often appear normal at birth and somatic signs usually start between two and four years of age [5,6]. The severity of this disease is broad, from mild to severe manifestations, with significant heterogenicity. The main distinction between the two types of manifestations is associated with central nervous system involvement, mainly represented by cognitive impairment and behavioral problems. Up to 2/3 of MPS type II affected patients have neurological impairment [3,7]. Individuals with mild disease have a minimal neurological deficit and can reach adulthood, but still show the disease’s other characteristics [8].

Clinical signs and symptoms include coarse facial features, short neck, and large head with growth retardation (resulting in short stature of the disproportionate type characterized by a short trunk) associated with skeletal deformities (dysostosis multiplex), contractures, joint stiffness, and carpal tunnel syndrome [6,7,9,10]. Respiratory involvement presents as recurrent upper and lower respiratory tract infections and sleep apnoea [3,10] and cardiac impairment with cardiomyopathy, mitral or aortic stenosis, and regurgitation [6,11] are common. Patients may also suffer from hearing loss, frequent ear infections, as well as retinal deterioration. Organomegalies (mainly of the liver and spleen) also occur [5,8]. Neurological involvement as hydrocephalus, cerebral atrophy, and cognitive impairment is seen in severe forms [7].

If left untreated, cardiorespiratory failure is commonly the cause of death by 10 to 15 years of age [7] in the severe forms, but those with mild forms can survive until beyond the fifth decade of life [4]. Treatment for MPS type II is directed at treating symptoms but, since 2006, enzyme replacement therapy (ERT) is also available with proven improvements in some somatic manifestations, such as improved forced vital capacity on pulmonary function tests (PFTs), decreased size of organomegalies, and an improvement on the six-minute walk test (6MWT) distance [5]. This evidence suggests that life expectancy may be increased by ERT. Early initiation of ERT has a great effect on some tissues that are more difficult to reach later on, like bones and cardiac valves. Ideally, treatment should start as soon as possible; however, an early diagnosis is rare for MPS type II, so therapy usually only starts when alterations are almost irreversible [6].

## Case presentation

**Figure 1:**
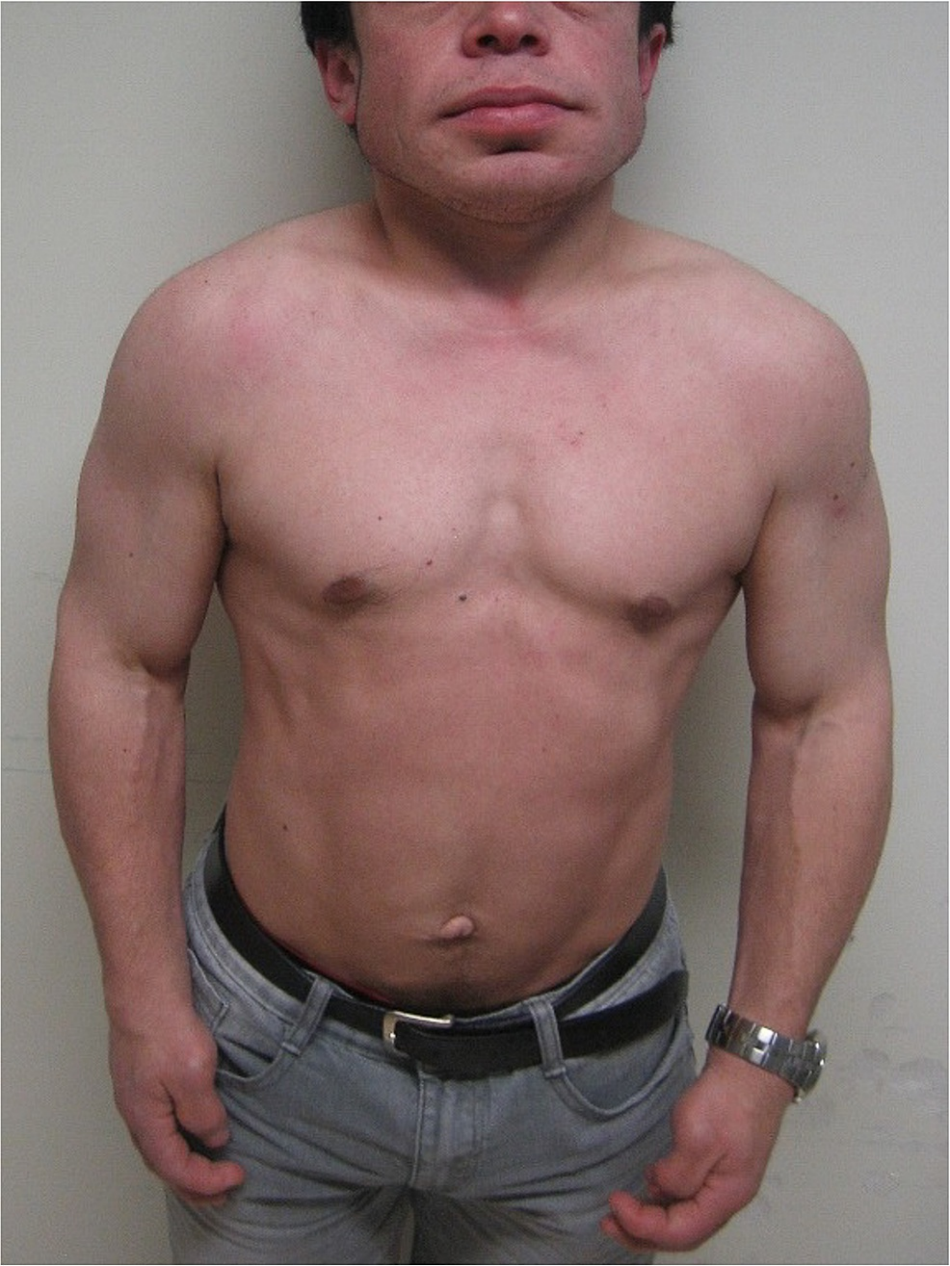
This figure shows our patient who has mild Hunter syndrome with coarse facial features and a large head. The neck is short, the nose is broad with a flattened bridge, and the lips are thickened. Short stature is also observed.

**Figure 2:**
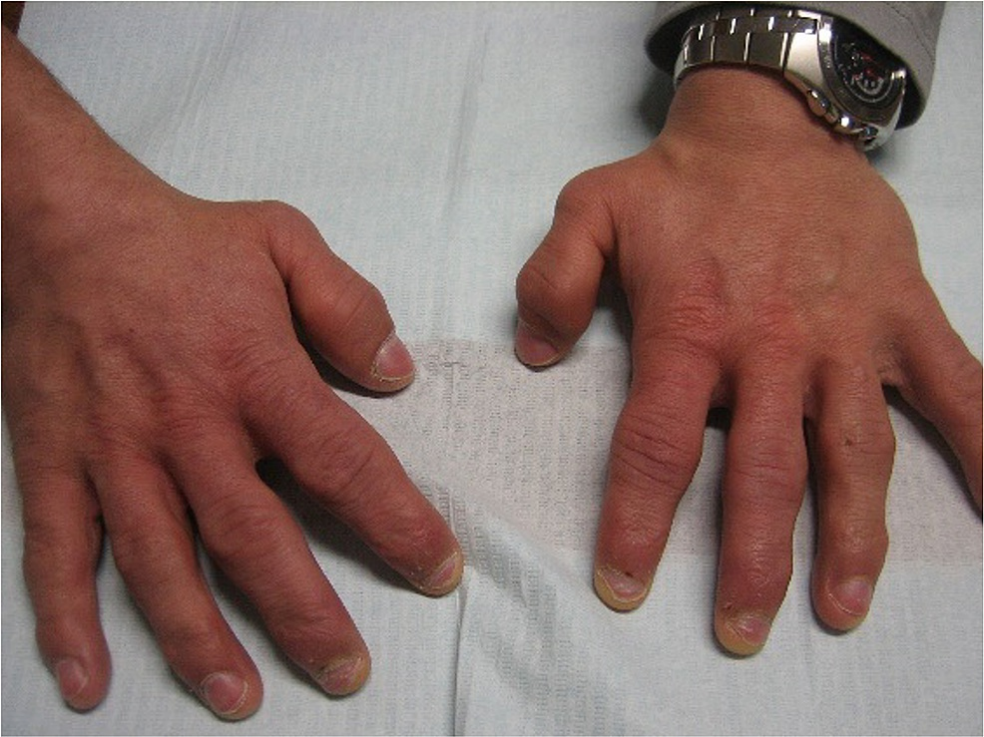
Our patient has broad thickened hands with stubby digits.

**Figure 3:**
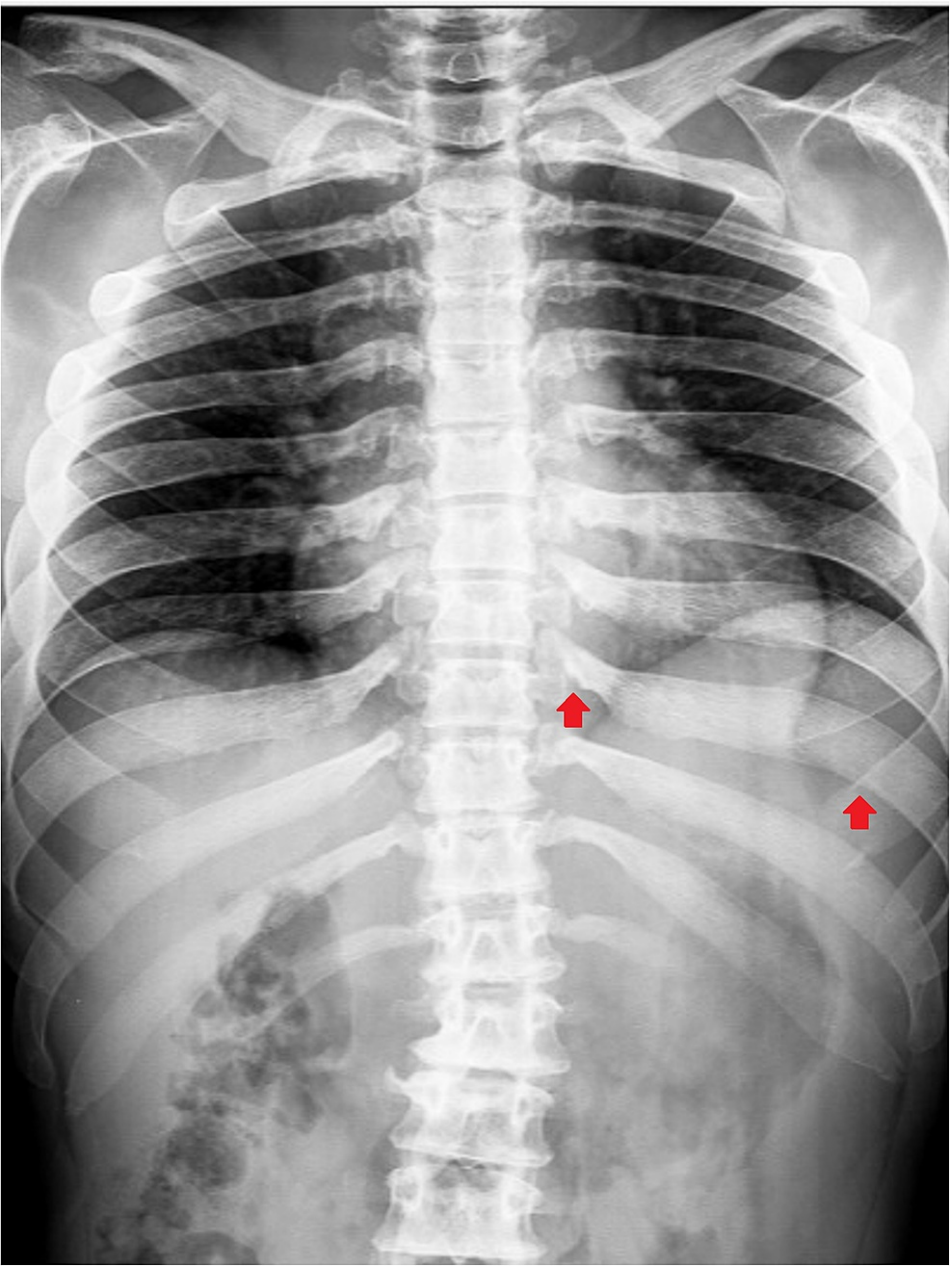
Plain thoracic and abdominal X-ray of our patient showing dysostosis multiplex manifested as ribs broadened distally and narrowed at the takeoff from the vertebral bodies, resulting in the oar shape (red arrow).

**Figure 4:**
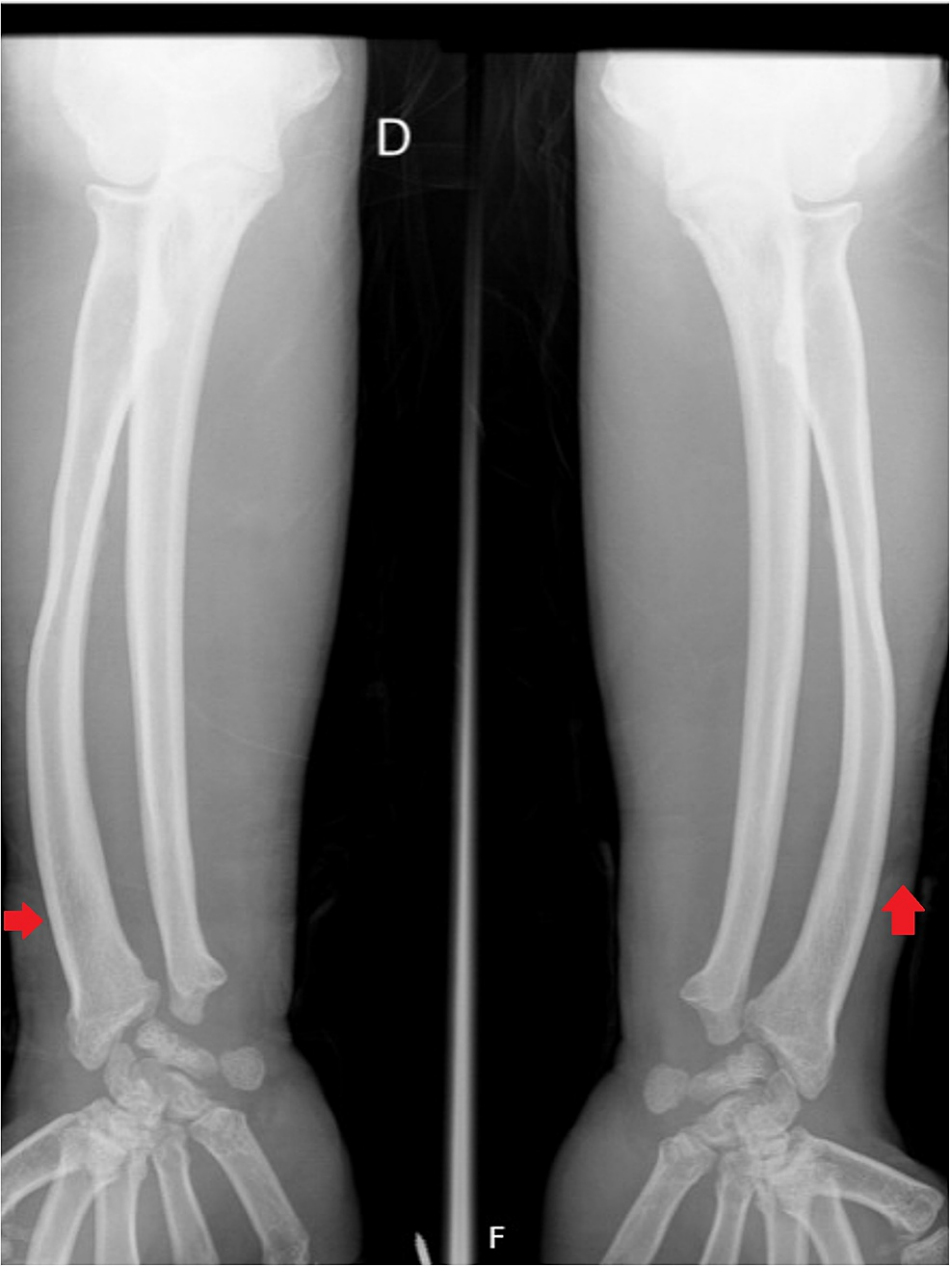
Plain X-ray of our patient's arms with dysostosis multiplex manifested as distal radius and ulna with abnormal angulation and tilting of the distal epiphyses (red arrow).

A 36-year-old man with no relevant family history was diagnosed with Hunter syndrome at nine years of age (with iduronate sulfatase level of 0.0 mmol/L/ml (normal range 105-262) and urinary GAG level of 42 mg/mmol creatinine (normal range 1-5)). He was referenced to our center at the age of 23, presenting symptoms such as short stature (1.42 m), short limbs, enlarged head, coarse facial features (broad nose and lower face with flared nostrils, thickened lips, irregular, and peg-shaped teeth) (Figure 1), broad thickened hands (Figure 2) with stubby digits, and dysostosis multiplex (Figures 3, 4).

**Figure 5:**
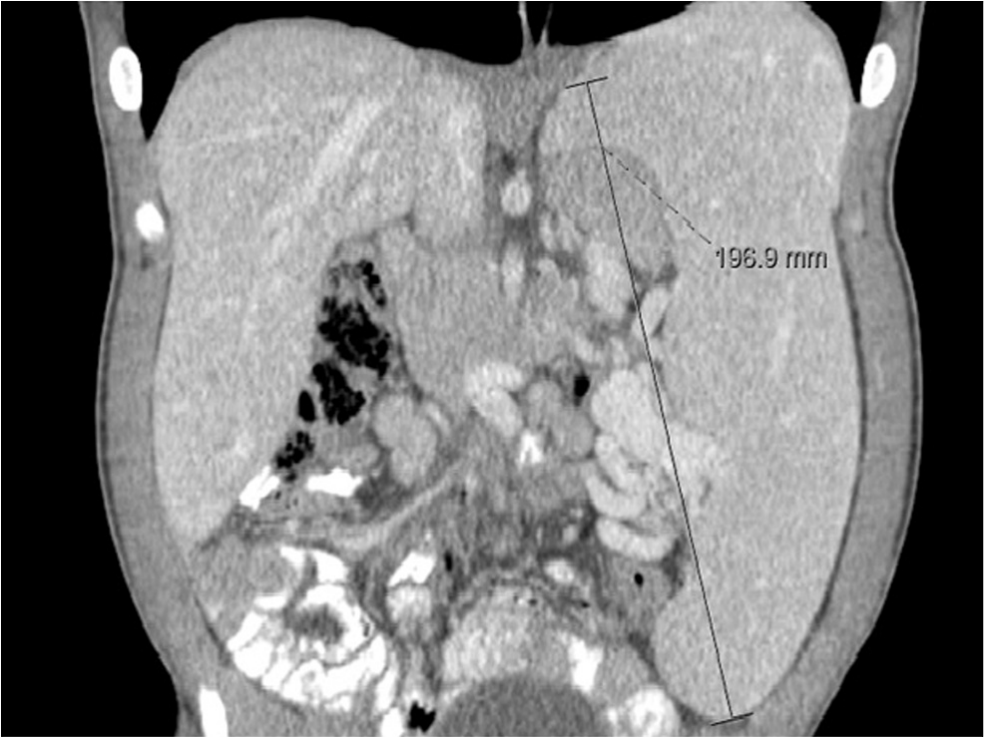
Abdominal CT-scan showing massive hepatosplenomegaly seen in our patient before treatment.

Regarding other involvements, a moderate obstructive ventilatory pattern was shown in PFT, in 6MWT, 526 meters were walked (representing 92% of the expected distance), with a minimum peripheral oxygen saturation of 92%, and echocardiography revealed mild aortic valve regurgitation. The patient presented a normal cognitive function (Mini-Mental State Examination 30/30) with no decline over the years. He had severe carpal tunnel syndrome for which he needed surgery. He also presented with major hepatosplenomegaly (Figure 5) with a liver volume of 1,728 cm^3^ (average liver volume was 1,450 cm^3^) and a spleen volume of 1,230 cm^3^ (average spleen volume was 400 cm^3^). Consequently, he had pancytopenia. He started ERT (at 23 years old) with a weekly injection of idursulfase (0.5 mg/kg) with good tolerance till now. During follow-up, there was a reduction of organomegalies with 33% and 55% decreases in the liver and spleen volumes, respectively. Along with this finding, cytopenias were resolved, urinary GAGs excretion diminished to 7 mg/mmol creatinine, and the patient repeated the 6MWT with 574 meters walked (96% of expected distance) with improved peripheral oxygen saturation (minimum 97%). He keeps an active life, works daily, and drives his car.

## Discussion

Classically, MPS is recognized as a children’s disease and, as such, the reports and particularities of the follow-up in adults are less explored. In recent decades, an increase in the number of adult patients has been observed, probably due to greater recognition of the disease and earlier diagnosis allowing earlier initiation of ERT [12]. The authors present this case since it is a rare disease, but also because it is less acknowledged in adults and its manifestations are so diverse that can lead to an evaluation by a particular specialty [12]. So, it is very important to recognize the systemic manifestations and not just one organ involvement to suspect an inborn error of metabolism.

There are 11 types of MPS, each one with different degrees of involvement, but MPS type II is the only type of MPS with X-linked inheritance [2,5,6,9]. Although rare, there are some women reportedly affected presenting with an attenuated form [13]. Loss of lysosomal enzyme iduronate-2-sulfatase activity in MPS type II leads to progressive lysosomal storage of undegraded GAGs, mainly heparan sulfate and dermatan sulfate in tissues and organs such as the liver, spleen, heart valves, bone, joints, and airways.

Infants with this disease appear normal at birth and developmental delays generally become apparent by 18-24 months of age [2,5], although before this age, affected patients are often initially taller than their peers, before decline growth rates [10]. Skeletal abnormalities of MPS type II patients are quite similar, despite the two clinical described forms. Coarsening of facial features becomes apparent usually by two to four years [2,5] of age, most likely due to a combination of bone dysostosis and GAG storage in the soft tissues of the orofacial region. Collectively known as dysostosis multiplex [6,14], the skeletal deformities of MPS type II include an abnormal thickness of all bones, irregular epiphyseal ossification in the hand, shoulder, and elbow joints, which results in joint stiffness and a claw-like appearance of the hands, enlarged clavicles, thickened and misshapen ribs, and vertebral irregularities. Carpal tunnel syndrome is believed to be related to the thickening of the ligaments and synovia, secondary to dysfunctional macrophages and fibroblasts associated with radio-ulnar dissociation [15].

MPS screening is made by quantitative measurement of urinary GAGs followed by electrophoresis to distinguish the GAG type [2]. Definitive diagnosis requires an assay of enzyme activity performed in leukocytes, fibroblasts, or plasma [3,6].

There is no curative treatment for these patients but with the advent of ERT, not only a delay in the disease's progression was seen, but also an improvement in some previously established organ damage was noted, as observed in our patient.

Treatment with ERT is indicated for all symptomatic patients with MPS type II regardless of the time of diagnosis; however, it is not recommended in patients with severe neurological impairment [16]. The treatment consists of weekly idursulfase injections, which in most countries implicates a weekly trip to a hospital, as long as the patient lives. The most reported side effects are associated with the infusion but otherwise is a well-tolerated treatment [9]. ERT showed a reduction in GAG levels and organomegalies [9] but limited efficacy was seen in some tissues like bone, cartilage, and cardiac valves. In these tissues, low bioavailability of the therapeutic enzyme is related to low vascularization and the presence of biological barriers, such as the blood-brain barrier for CNS treatment [17]. Since our patient had a normal cognitive function, treatment restrictions were less limiting.

Periodical assessment of blood lineages, organ volumes, and respiratory function is made to evaluate response to treatment and early recognition of complications. During the 13 years of follow-up since our patient started treatment, ERT proved to be effective not only by reducing organomegalies with secondary improvement in blood lineages but also in stabilizing the disease allowing the patient to lead a good active life. No complications were reported.

And even though ERT is a great advance for the treatment of these patients, some manifestations need follow-up from several specialties. Treating these patients in reference centers, with multidisciplinary teams, offers treatment with knowledge, accounting for all the affected organs, improving their quality of life and prognosis. In the presence of bone and cartilage lesions, sometimes with marked deformation, a physiatrist and orthopedist are of extreme importance to the multidisciplinary team. Our patient presented carpal tunnel syndrome and he was promptly treated by orthopedics. Recurrent lower respiratory tract infections with the potential to be serious conditions, especially when associated with bone deformities of the thoracic cavity, and ear infections show the importance of having an internal medicine doctor, pulmonologist, and otolaryngologist on the team. Since the neurological manifestations with important cognitive impairment are prevalent in patients with MPS type II (not the case of our patient), the integration of a neurologist in the team is essential. Transition programs from pediatric centers are essential to ensure the patient and his family continuousness of differentiated care.

## Conclusions

MPS type II is a rare heterogenic and multisystemic disorder. This case is an example of a mild form of MPS type II, with musculoskeletal, cardiac, and respiratory features, but with no cognitive impairment. Early disease identification and phenotype recognition are the keys to better care. Genetic counseling for the patient and the family is very important as it can identify reproductive risks, provide prenatal or preconception options, and even early referral to a reference center. An integrated and multidisciplinary approach, as well as accurate surveillance on new possible signs of the disease, is necessary. Though no curative treatment is available, ERT changes the disease’s outcome and improves the global functional ability, as seen in this patient, even started (later) in cases with relevant organ involvement. ERT resulted in substantial reductions in hepatosplenomegaly and urinary GAG excretion, indicating efficient clearance of lysosomal GAG.
